# Cross-Kingdom Communication via Plant-Derived Extracellular Vesicle Nucleic Acids in Genetically Engineered *Nicotiana tabacum*

**DOI:** 10.3390/genes16030356

**Published:** 2025-03-20

**Authors:** Lorena Urbanelli, Federica Delo, Giada Cerrotti, Emidio Albertini, Jacopo Lucci, Sandra Buratta, Eleonora Calzoni, Stefano Giovagnoli, Luana Lugini, Cristina Federici, Federica Fratini, Valentino Mercati, Carla Emiliani

**Affiliations:** 1 Department of Chemistry, Biology and Biotechnology, University of Perugia, Via del Giochetto, 06123 Perugia, Italy; lorena.urbanelli@unipg.it (L.U.); giada.cerrotti@dottorandi.unipg.it (G.C.); sandra.buratta@unipg.it (S.B.); eleonora.calzoni@unipg.it (E.C.); 2 Centro di Eccellenza sui Materiali Innovativi Nanostrutturati (CEMIN), University of Perugia, Via del Giochetto, 06123 Perugia, Italy; 3 Bios-Therapy, Physiological Systems for Health S.p.A., Loc. Aboca 20, Sansepolcro, 52037 Arezzo, Italy; fdelo@biostherapy.it (F.D.); jlucci@biostherapy.it (J.L.);; 4 Department of Agricultural, Food and Environmental Sciences, University of Perugia, Borgo XX Giugno 74, 06121 Perugia, Italy; emidio.albertini@unipg.it; 5 Department of Pharmaceutical Sciences, University of Perugia, 06126 Perugia, Italy; stefano.giovagnoli@unipg.it; 6 Istituto Superiore di Sanità, Viale Regina Elena, 299, 00161 Rome, Italy; luana.lugini@iss.it (L.L.); cristina.federici@iss.it (C.F.); federica.fratini@iss.it (F.F.)

**Keywords:** *Nicotiana tabacum*, plant-derived extracellular vesicles, cross-kingdom communication, extracellular RNA

## Abstract

Background/Objectives: Plants release extracellularly lipid bilayer-enclosed vesicles of nanometric size that can be retrieved in their fluids. Plant-derived extracellular vesicles (PDEVs) have mostly been involved in modulating host–pathogen interaction, making them a tool for cross-kingdom communication with a key role in plant immunity. In addition, PDEVs have demonstrated promising therapeutic features, not only in terms of intrinsic nutraceutical properties but also of active molecules’ delivery. Transgenic plants have been developed for a variety of purposes, i.e., to improve their functional properties like crops, but also to produce therapeutic molecules. However, it is unclear whether transgenes can end up in PDEVs, thus making them a vehicle for their cross-kingdom diffusion into the environment. Methods: Here, we investigated the association of transgenic DNA and RNA with PDEVs secreted by tobacco (*Nicotiana tabacum*) engineered to express the neomycine phosphotransferase II (Npt-II) gene. PDEVs were isolated from leaf apoplastic fluid by ultracentrifugation and characterized for their morphology and size. The association of DNA and RNA was assessed by qRT-PCR and their immunomodulatory properties by assaying PDEVs-induced IL1β and IL10 on THP1 monocytes. Results: Npt-II RNA, but not DNA, could be amplified from PDEVs, whereas no differences were observed between wt and transgenic tobacco PDEVs in terms of immunomodulatory properties. Conclusions: Although a different behaviour by other types of RNAs or DNAs could still be possible, our findings indicate that in this model, PDEVs are not associated with transgenic DNA, but they can protect RNA, including transgenic RNA, from degradation, contributing to their cross-kingdom spreading.

## 1. Introduction

Plant-derived extracellular vesicles (PDEVs) are membrane-surrounded particles of nanometric size (50–500 nm) [1,2]. These vesicles exert many functions in plant cells, although nowadays their most characterized biological role is related to the host response against pathogens, namely fungi, but also bacteria and viruses [3,4]. As they are relatively stable and able to protect their biochemical content from extracellular environment insults, PDEVs are a tool for cross-kingdom intercellular communication, between plant and animal cells and between plants, or animals, and microorganisms [5,6]. In addition to their role in plant immunity, it must be considered that PDEVs are one of the vegetal components ingested with edible plants [7]. Consequently, they may contain relevant biomolecules reminiscent of the releasing cells and are being investigated as oral supplements for nutritional purposes, either for their intrinsic properties, or as nanocarriers of other compounds loaded into vesicles following their isolation. In this context, PDEVs appear to be an economic source of particles, with less safety issues with respect to those originating from animal sources.

PDEVs contains proteins, lipids, and nucleic acids. Among nucleic acids, the presence of different types of RNA has been reported, whereas the presence of DNA has not been investigated in plant vesicles but has been reported in mammalian EVs [1,8]. Indeed, mitochondrial DNA, as well genomic DNA, including oncogenes, has been retrieved in EVs released by cultured cells and biological fluids [9,10]. PDEVs contain different types of RNA, such as small interfering RNAs (siRNAs) and microRNAs (miRNAs), and it has been demonstrated that the biological effect on target cells may be mediated by RNA, as small RNAs vehiculated into fungal pathogens are able to suppress infection via RNA interference (RNAi) [11]. It is unclear whether PDEVs can vehiculate relatively long mRNA.

Transgenic plants are characterized by the modification of DNA using recombinant DNA technology, leading to the insertion of a gene sequence known as transgene, coming from another plant or a different source [12,13]. The purpose is to obtain plants with improved properties in terms of crop properties (yield, quality) and plant resistance to stress, but more specialized applications have been developed, such as the use of transgenic plants as bio factories for the economic production of high value-added products, such as recombinant proteins, including antibodies [13]. The use of recombinant DNA technology to enhance the crop content of specific biomolecules, such as metabolites and micronutrients, must also deal with the assessment of their environmental risk. Indeed, the transgene DNA may possibly spread to non-target organisms such as pollinators, as well as herbivores and soil organisms [14]. More specifically, a large concern has regarded the possible wide cross-kingdom diffusion of selectable marker genes (SMGs) represented by bacterial antibiotic resistance genes [15]. Although the introduction of gene editing techniques has changed perspective, making it relatively easy to edit plants without the need of SMGs, many aspects of nucleic acids’ diffusion into the environment remain to be elucidated [16].

In this context, the finding that PDEVs represent a tool for cross-kingdom communication has raised the question of whether they could contribute to the spreading of transgene, such as those encoding for antibiotic resistance in the environment, namely pathogenic microorganisms, but also herbivores and other animals. Based on these observations, we decided to investigate the presence of transgenic DNA and RNA associated with PDEVs isolated from leaves of tobacco (*N. tabacum*) genetically modified to express neomycine phosphotransferase II (Npt-II) gene [17,18]. We isolated EVs by differential centrifugation and obtained evidence that the mRNA of the transgene, but not DNA, may co-isolate with PDVEs. Although different behaviour by other types of RNAs or DNAs could still be possible, our results provide evidence that PDEVs may be associated with and protect from degradation relatively long mRNA, thus representing a possible vehicle of cross-kingdom diffusion of the transgene in genetically engineered plants.

## 2. Materials and Methods

### 2.1. Isolation of Plant Extracellular Vesicles from Apoplastic Fluid

Plant growth took place in a greenhouse in spring, under natural sunlight, temperature, humidity, and CO_2_. Growth conditions followed Legislative Decree 227/2016 regulating the cultivation of genetically modified organisms on the territory of Italy. Seeded plants were allowed to grow for at least 8 weeks. PDEVs were prepared according to Rutter and Innes [19], with minor modifications. Briefly, leaves were harvested, washed in water, and dried by blotting excess water on filter papers. They were weighed and rolled around a 1000 tip and inserted into a 60 mL syringe with Vesicle Infiltration Buffer (VIB) (20 mM MES, 2 mM CaCl_2_, 0.1 M NaCl, pH 6.0). The isolation of PDEVs from apoplastic fluid was performed by differential ultracentrifugation (dUC). To recover the apoplastic liquid, the leaves, rolled on tips, were centrifuged at a low speed (700× *g* for 20 min at 4 °C), and then the recovered liquid was filtered at 0.45 µm to remove any debris. Subsequently, the recovered liquid was subjected to centrifugation steps at increasing speed (10,000× *g* for 10 min, 40,000× *g* for 60 min, 100,000× *g* for 60 min). The fractions recovered, in accordance with the protocol, were the pellet after centrifugation at 40,000× *g* (40 K fraction) and 100,000× *g* (100 K fraction). Pellets were washed in VIB and pelleted at the same speed. EVs were then resuspended in a volume of 100 µL of VIB and stored at −80 °C, unless differently indicated. All reagents were of analytical grade and obtained from Merck Life Science (Darmstadt, Germany).

### 2.2. DNA Extraction and PCR

DNA from leaf tissue or EVs was extracted and purified with the PureLink Plant total DNA Purification Kit (Thermo Fisher Scientific, Waltham, MA, USA). DNA was eluted in 30 µL of Elution Buffer and quantified with a spectrophotometer (Bio-Photometer, Eppendorf, Hamburg, Germany). PCR was carried out using Dream Taq DNA polymerase (Thermo Fisher Scientific). PCR reactions were carried out with the Thermo Hybaid PCR Express System (Thermo Fisher Scientific). PCR amplifications of the gene NPTII were carried out with NPTII.for 5′-TCG GCT ATG ACT GGG CAC AA and NPTII.rev 5′-CCTTGAGCCTGGCGAACAGT. It produced an amplicon of 468 bp. ITS and RPS were amplified with ITS.for 5′-TCC TCC GCT TAT TGA TAT GC/ITS.rev 5′-GGA AGT AAA AGT CGT AAC AAG G and RPS. For 5′-GGT GCG ACT TTG GTA GAA AGC AAC/RPS.rev 5′-TCG GGA TCG AAC ATC AAT TGC AAC, producing amplicons of 738 bp and around 918 bp, respectively. PCR products were subjected to electrophoresis in a 1.5% agarose gel.

### 2.3. RNA Extraction, Reverse Transcription, and RT-qPCR

Approximately 400 mg of leaf was taken, frozen in liquid nitrogen, and moved to −80 °C. The leaf was then shredded with a grinder pre-cooled at −80 °C to obtain small pieces without thawing the tissue. This step was introduced to reduce the release of RNases, which compromised extraction. Leaf tissues were placed in a 2 mL tube, and RNA was extracted either with EuroGold Trifast (EuroClone, Pero, Italy) or with the mirVana miRNA isolation kit (Thermo Fisher Scientific). In the first case, 200 μL of chloroform was added to 1 mL of sample solubilized in Trifast, and then the mixture was vortexed and centrifuged at 12,000× *g* for 15 min at 4 °C. The aqueous phase was moved into a new tube containing 0.5 mL of isopropanol per mL of Trifast and centrifuged at 12,000× *g* for 15 min at 4 °C. After centrifugation, the supernatant was discarded and the RNA pellet was resuspended in 1 mL of 75% ethanol and then centrifuged at 7500× *g* for 5 min at 4 °C. The supernatant was discarded and the pellet was air-dried and then finally resuspended in 20 μL of nuclease-free water. In the second case, the MirVana miRNA isolation kit, which allows for the purification of total RNA with particular attention paid to low-molecular-weight RNAs, was used according to the manufacturer’s instructions. The same protocols were used for the extraction of total RNA from the 100 K fraction of vesicles from apoplastic fluid. In this case, the vesicles were thawed from −80 °C and Trifast (100 µL per 20 µL of 100 K vesicle suspension) or mirVana Lysis/Binding Buffer (100 µL per 20 µL of 100 K vesicle suspension) was added to vesicles isolated from about 5.0 g of apoplastic fluid (corresponding to about 12.5 g of starting leaves). RNA was quantified by measuring absorbance at 260 nm. A fixed volume of RNA was reverse-transcribed using the Maxima H Minus First Strand cDNA Synthesis Kit (Thermo Scientific) at a total volume of 20 μL to produce first-strand cDNA. qPCR was carried out on the diluted 1:10 cDNA as the template and the NPTII gene primers pair. The reaction was performed in triplicate using the SYBR Green Master Mix (Bio-Rad, Hercules, CA, USA) following the manufacturer’s protocol using a StepOnePlus thermocycler (Applied Biosystems, Foster City, CA, USA).

### 2.4. SEM Imaging (Scanning Electron Microscopy)

PDEVs were isolated as described in 2.1 and then prepared for SEM analysis as previously described [20]. Briefly, 100 K and 40 K fractions were fixed in glutaraldehyde to prepare for SEM. Vesicles were resuspended in approximately 30 µL of VIB (Buffer VIB filtered 0.22 µm), and proteins were quantified by the Bradford method (Quick Start Bradford assay, Bio-Rad). The 40 K fraction protein amount was too low to be quantified. A total of 2 µg of proteins of the 100 K EVs sample and an arbitrary volume of 40 K EVs (2/3 of the sample) were used. Vesicle samples were diluted in 2 mL of 2.5% glutaraldehyde (*v*/*v*) in Phosphate-Buffered Saline (PBS) 1X and fixed for 15 min. Then, they were washed in 15 mL of dd-water (0.22 µm filtered), placed in Vivaspin (Sartorius, Göttingen, Germany) tubes (cut off 300 KDa), and centrifuged at 3000× *g* for 3 min. The eluate was discarded and samples were washed again twice in 15 mL of water and centrifuged under the same conditions. Finally, concentrated EVs were recovered and moved to 1.5 mL tubes. Two dilutions were prepared (1:20 and 1:500 of the recovered vesicles), and 20 µL of each dilution was seeded onto 12 mm diameter glass coverslips. The slides were fixed on a metal support and metallized with a thin layer of conductive material for electron microscopy.

### 2.5. NTA (Nanoparticle Tracking Analysis)

PDEVs (100 K fraction), isolated as described in 2.1, were resuspended in PBS at a suitable concentration for particle count (2 × 10^8^–1 × 10^9^ particles per mL) and vortexed for 1 min. The NS300 instrument (Malvern Panalytical, Malvern, UK) was used to analyze the samples. The analysis processed 5 × 60 s × 25 frames/s for each sample, using NTA 3.4 analytical software build 3.4.4. Particle size distribution was determined by reporting the percentage of particles with the specified diameter relative to all of the particles measured. All solutions used were previously filtered through a 0.22 μm filter.

### 2.6. Cell Stimulations and Treatments

The human monocytic leukaemia cell line THP-1 (ATCC TIB-202) was grown in Roswell Park Memorial Institute (RPMI) 1640 culture medium supplemented with foetal bovine serum and penicillin/streptomycin (P/S) to 10% and 1%, respectively, at 37 °C in 5% CO_2_ in a humidified incubator. THP-1 cells were seeded in a 6-well plate (1 × 10^6^ cells/well). In one well, the mature macrophage-like state was induced by treating THP-1 monocytes for 24 h with 1 µg of LPS (Cell Signaling Technology, Danvers, MA, USA) directly added to growth medium. The 100 K EVs fraction was isolated, both from wild-type and transgenic tobacco leaves, as described in Section 2.1. The final pellet was then resuspended in 100 µL of PBS, and 10 µL was used to assay the protein concentration using the Bradford method. Cells were treated with two different concentrations of EVs for 24 h: in one well, approximately 0.8 µg/mL was added to the EV-depleted medium, and in another well, 2.4 µg/mL was added. The procedure was repeated both for PDEVs from wild-type and transgenic tobacco leaves. Following treatment with either LPS or PDEVs for 24 h, RNA was isolated using the Trizol method. cDNA was synthesized from 1 μg of total RNA using the SuperScript IV First-Strand Synthesis System (Thermo Fisher Scientific). About 700 ng of cDNA was used for qRT-PCR. Glyceraldehyde-3-phosphate dehydrogenase (GAPDH) was chosen for normalization because this gene was stably expressed in both THP-1 monocytes and macrophages. The reaction was performed in triplicate, and the PCR products of all samples were subjected to a melting curve analysis to verify the single amplification product. The mRNA expression relative to GAPDH was calculated by the ΔCt method, and then ΔΔCt was determined to express the fold change in each of the primer pairs, calculated as 2^−ΔΔCt^. The following primer pairs were used: GAPDH for 5′- TGC ACC ACC AAC TGC TTA GC and GAPDH.rev 5′- GGC ATG GAC TGT GGT CAT GAG; IL-10.for 5′-GTG ATG CCC CAA GCT GAG A and IL-10.rev 5′- CAC GGC CTT GCT CTT GTT TT, IL-1β.for 5′-GTG GCA ATG AGG ATG ACT TGT TC and IL-1β.REV 5′- TAG TGG TGG TCG GAG ATT CGT A.

## 3. Results

### 3.1. Experimental Model and PDEVs Isolation

**Figure 1:**
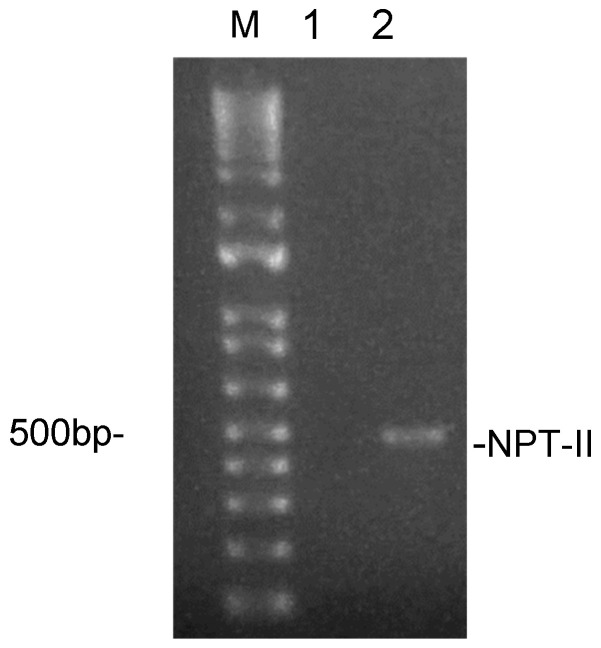
Analysis of the NPT-II gene in tobacco leaves. DNA was extracted from leaves, amplified by PCR with NPT-II.for and NPT-II.rev primers, and run on 1.5% agarose gel. Lanes 1 is a negative control, loaded with the amplification product from wt tobacco, while lane 2 is loaded with the amplification product of Npt-II tobacco. Lane M, 100 bp DNA ladder.

Tobacco (*N. tabacum*) plants expressing a conventional Npt-II kanamycin selection system (NPT-II transgenic tobacco, TG) were previously described [17,18]. The presence of the NPT-II transgene was confirmed by PCR using DNA extracted from leaves as the template. Indeed, no amplification could be obtained by wild-type tobacco (WT) genomic DNA (Figure 1).

PDEVs were isolated from the apoplastic fluid of NPT-II transgenic tobacco leaves to determine whether it was possible to amplify transgenic DNA from these vesicles. Leaves were washed, dried, and weighed and then PDEVs were isolated as described in Section 2.1. The apoplastic fluid (approximately 1 g for 2.5 g of leaf weight) was filtered and centrifuged at 10,000× *g* and then the supernatant was recovered and centrifuged again at 40,000× *g*. The pellet was resuspended in buffer (40 K fraction), while the supernatant was recovered and ultracentrifuged at 100,000× *g*. Following the ultracentrifugation step, the pellet was resuspended in buffer (100 K fraction), while the supernatant was discarded. As determined by the protein concentration assay, the procedure allowed us to recover, for 1 g of apoplastic fluid, about 1 µg of PDEVs for the 40 K fraction and 3.5 µg of PDEVs for the 100 K fraction. To confirm the presence of PDEVs in the 40 K and 100 K fractions, we carried out morphological analysis by SEM. Both fractions contained round-shaped vesicular structures with a heterogenous size, with the 40 K fraction characterized by the presence of aggregates.

**Figure 2:**
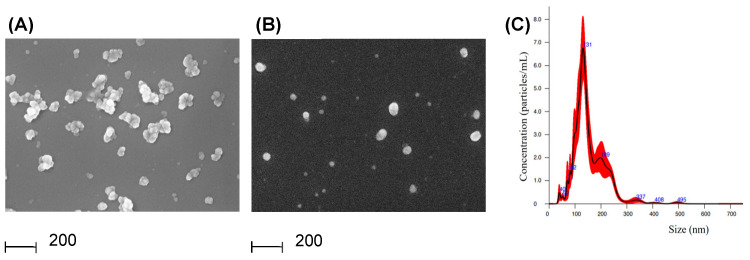
Characterization of PDEVs released from Npt-II tobacco. (**A**,**B**) Scanning electron microscopy images of PDEVs. Samples were fixed using 2.5% glutaraldehyde and then allowed to dry at room temperature onto glass coverslips. (**A**) PDEVs 40 K fraction; (**B**) PDEVs 100 K fraction. (**C**) Nanoparticle tracking analysis of PDEVs of the 100 K fraction. Samples were resuspended in 0.22 μm filtered PBS and then loaded into a NS300 Malvern instrument. Data are reported as particles/mL, with respect to size.

Therefore, we carried out Nanoparticles Tracking Analysis (NTA) only of the 100 K fraction to gain insight into PDEVs’ size distribution. The results provided evidence that PDEVs had a mean size of 154.3 ± 6.9 nm, with a mode size of 128.0 ± 5.6 nm (Figure 2C). No significant differences in terms of yield, morphology, or size distribution were observed when PDEVs were isolated from the leaf apoplastic fluid of wt.

### 3.2. Characterization of Nucleic Acids’ Association with PDEVs

To detect the presence of the Npt-II transgene DNA in PDEVs, 20 g of leaves was used to isolate PDEVs, and all the recovered 100 K fraction vesicles were used to extract DNA. End-point PCR was carried out in the same conditions reported above to assess the presence of the transgene in DNA extracted from leaves, and the amplification mix was loaded onto 1.5% agarose gel. However, it was not possible to detect any amplification from DNA extracted from PDEVs isolated from Npt-II tobacco. Similar results were obtained when the sensitivity of the system was increased by the qRT-PCR method; further, in this case, no amplification could be detected when 100 K fraction PDEVs were used as the DNA source, whereas amplification could be detected when DNA was extracted from the leaf, with a cycle threshold of 29.74 ± 0.8 and a melting temperature (T_m_) of 89.1 ± 0.1 °C. For this reason, we concluded that genomic DNA, including the transgene, was not efficiently packed and/or associated with 100 K fraction PDEVs.

To complete the analysis of the transgene nucleic acid association with PDEVs, we then determined whether PDEVs may carry mRNA encoding the transgene. We isolated RNA from 100 K fraction PDEVs contained in the apoplastic fluid of ~15 g of leaves belonging either to Npt-II tobacco, using two different reagents, i.e., EuroGold Trifast (EuroClone), or the mirVana miRNA isolation kit (Thermo Fisher Scientific). cDNA was then used using the more sensitive qRT-PCR analytical approach. As it is possible to observe (Table 1) from both leaf RNA, representing a positive control, and RNA obtained from the 100 K fraction of PDEVs using either Trifast or miRVana, amplifications of good technical quality could be obtained.

To confirm that the amplicons from leaf and/or PDEVs (100 K fraction) were identical, we analyzed their denaturation curves, which showed an identical temperature of melting ™, i.e., 89.02 °C, thus confirming that the same amplification product was obtained using as the template either leaf or PDEVs (100 K fraction). In addition, similar results were obtained when qRT-PCR was carried out using mRNA extracted with Trifast (method 1) or miRVAna (method 2). Of course, no amplification was obtained in the absence of template cDNA. The successful amplification of NPT-II mRNA from PDEV of the 100 K fraction indicated that the mRNA was associated with PDEVs, and this association protects it from undergoing degradation.

**Figure 3:**
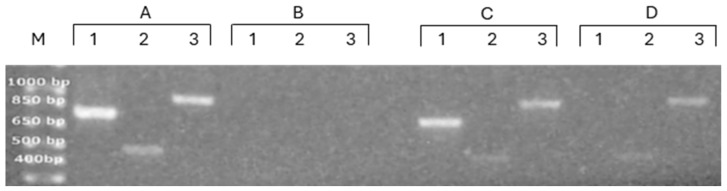
Analysis of the ITS, Npt-II, and rps amplicons in DNA and RNA isolated from leaf and 100 K fraction PDEVs of Npt-II tobacco. DNA was extracted from leaf (indicated with A) or from 100 K fraction PDEVs (indicated with B) isolated from the apoplastic fluid obtained from 15 g of leaves, amplified by PCR, and run on 1.5% agarose gel. cDNA was reverse-transcribed from RNA extracted from leaf (indicated with C) or from 100 K fraction PDEVs (indicated with D) isolated from the apoplastic fluid (~15 g of leaves), amplified by PCR, and run on 1.5% agarose gel. The ITS amplicon is indicated with 1, the Npt-II amplicon with B, and the rps amplicon with 3. Lane M, 100 bp DNA ladder.

After confirming that the NPT-II transcript was co-isolated with EVs from the 100 K fraction, we investigated whether other genes, which are encoded by tobacco, were also co-isolated with EVs. We examined the presence of two additional amplicons: the first one, named the ITS (Internal Transcribed Spacer), about 768 bp length, is included in a region of the ribosomal RNA precursor, so it is a transcribed region that is not removed during rRNA maturation. The second amplicon, named rps, about 918 bp length, encodes for the mitochondria/chloroplast small subunit; it is transcribed using these organelles DNA as the template and is not part of nuclear genomic DNA. Therefore, many copies are present in the sample. Figure 3 shows that ITS, NPT-II, and rps amplicons could be obtained using as the template DNA from leaf but not DNA from PDEVs (Figure 3, lanes A and B, respectively). Similarly, all three amplicons could be detected when cDNA obtained from leaf RNA was used as the template (Figure 3, lanes C), and, interestingly, signals could also be detected using end-point PCR when cDNA obtained from PDEVs RNA was used as the template, very clearly for the rps amplicon, less intense but detectable for the NPT-II gene, and not clearly detectable for the ITS amplicon. These results indicated that mRNA encoded by the mitochondrial genome, which is present in high copy numbers, can be detected as associated with PDEVs of the 100 K fraction, protected from degradation, whereas ITS, which is an RNA destined for degradation as it is not a part of mature ribosomal RNA, could not be detected in these conditions.

### 3.3. Gene Expression Kinetics of THP-1 Stimulated with LPS and EVs

**Figure 4:**
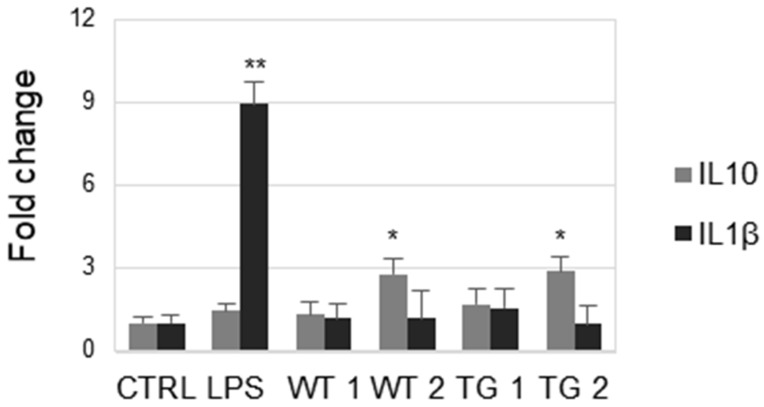
Gene expression of IL-10 and IL-1β in response to administration of PDEVs isolated either from wild-type (WT) or NPT-II transgenic (TG) tobacco. THP-1 cells were stimulated for 24 h with approximately 0.8 µg (WT 1 and TG 1) or 2.4 µg of PDEVs (WT2 and TG 2), diluted in 1 mL of cell culture medium. LPS (100 ng/mL) was used as control. Relative gene expressions with respect to untreated THP-1 cells (CTRL) are displayed. The analysis was repeated four times in triplicate. The mean ± SE is reported (* *p* < 0.05; ** *p* < 0.01).

Previous studies have provided evidence that PDEVs exert immunomodulatory effects in vitro and in vivo [21]. Therefore, we determined whether PDEV isolated from the apoplastic fluid of both wild-type and transgenic tobacco modulated inflammation in the in vitro cell model. THP-1 cells, like primary monocytes and macrophages, express a variety of inflammation-related cytokine genes in response to different stimuli. The expression of IL-1β, a pro-inflammatory cytokine [22], and IL-10, which is considered an anti-inflammatory cytokine [23], was assessed in THP-1 cells following the administration of PDEVs isolated from the apoplastic fluid of both wild-type and transgenic tobacco. THP-1 was incubated with two different concentrations of PDEVs (approximately 0.8 µg and 2.4 µg/mL), and then the mRNA expression level of IL-1β and IL-10 was determined by qRT-PCR (Figure 4). The levels of cytokines induced by the endotoxin lipopolysaccharide (LPS), which has been shown to promote cytokine release in various cell culture models [24], were assessed as the positive control. Gene expression changes were compared to those of untreated cells and analyzed as control. The results showed that the levels of the pro-inflammatory cytokine IL-1β are not increased in PDEV-treated cells compared to LPS-treated cells, but only in the positive control represented by LPS-treated cells. On the other hand, the anti-inflammatory cytokine IL-10 is instead increased (*p* < 0.05) in cells treated with the highest concentration of PDEVs, either isolated from wild-type or transgenic tobacco.

## 4. Discussion

PDEVs play an important role in mediating cell–cell communication and performing biological functions. They are implicated in host defence towards pathogens, as well as establishing and maintaining symbioses; for these purposes, their content in functional molecules, such as proteins, lipids, and nucleic acids, is fundamental [25,26], and PDEVs protect their biochemical content, thus making them mediators of cross-kingdom intercellular communication between plants and microbes [27]. PDEVs contain different types of nucleic acids, namely diverse species of small RNAs, such as miRNA and siRNA [28], but also other non-coding RNAs (Y-RNAs, rRNAs, tRNAs) and coding mRNAs fragmented and in full length [29,30,31]. So far, the presence of transgenic nucleic acid in association with PDEVs has not been specifically investigated.

We isolated PDEVs from transgenic tobacco plants carrying the selectable marker NPT-II by differential centrifugation, obtaining a 100 K fraction, showing the presence of EVs by both SEM and NTA. Using cDNA reverse-transcribed from the RNA contained in the EVs of the 100 K fraction, we succeeded in amplifying the NTP-II gene. However, we did not obtain a successful amplification when DNA extracted from the 100 K fraction EVs was used as the template. The detection of a large fragment of the transgene mRNA in association with EVs indicates that the vesicular fraction is protective toward RNA degradation in the extracellular environment, where RNA is usually rapidly subjected to degradation by RNAses.

We also succeeded in amplifying another mRNA, rps, which is transcribed from the mitochondrial genome. This finding clearly indicates that the 100 K vesicular fraction can protect mitochondrial RNA from degradation. Of course, the presence of a high copy number of mitochondrial DNA may favour amplification, but it is of relevance that mRNA from mitochondria genome transcription is also protected from degradation in the vesicular fraction. On the other hand, we did not observe amplification signals when we tried to amplify an ITS sequence. This sequence is contained is the spacer DNA localized between the small-subunit rRNA and the large-subunit rRNA sequences. Consequently, it is destined to degradation during the post-transcriptional maturation of rRNA, and therefore its half-life is brief. In this case, we did not observe any amplification, indicating that short half-life RNAs are not destinated to be protected from degradation by PDEVs [32]. Overall, our results clearly indicated the ability of PDEVs to package and/or associate, and then protect, not only small RNAs but also other RNAs such as mRNAs, with implications for horizontal gene transfer and cross-kingdom communication.

Among bioactive compounds in plants, our diet also includes nucleic acids. This dietary RNA content, particularly small RNAs, has raised considerable interest over recent years [33]. Indeed, there is the possibility of the penetration of plant miRNAs through the gastrointestinal (GI) barrier, which could enter the circulatory system in mammals and affect gene expression [33]. In 2012, a pioneering study by Zhang et al. [34] reported that miR168, a miRNA abundant in rice, could bind to the human/mouse low-density lipoprotein receptor adapter protein 1 mRNA, inhibit its expression in liver, and decrease LDL removal from mouse plasma. However, despite the increasing amount of data indicating the possibility of the penetration of miRNA from the diet, several authors experienced difficulties in replicating these results, and the evidence of cross-kingdom regulation by dietary RNA is still under debate [35,36].

Nevertheless, there is a lot of interest in dietary RNA, because the recent evidence that PDEVs can protect package and/or associate different types of RNA has raised the possibility that they could shuttle RNAs across different species belonging to different kingdoms, particularly in delivering plant RNA to recipient cells in the GI tract after oral intake. Apart from the interest in assessing the potential biological role of cross-kingdom communication mediated by PDEV RNAs, there is also considerable attention related to the potential applications of PDEVs RNAs cargo, either naturally present or artificially loaded, for the therapeutic delivery of RNAs, such as miRNAs. Indeed, these edible plant-derived vesicles are generally regarded to be safe. In addition, they possibly interact not only with GI tract cells but also with gut microbiota, thus suggesting the possibility that this interaction could be exploited to deliver therapeutic RNA-targeting microbes for therapeutic purposes.

In this context, the fact that transgenic plants are characterized by the modification of DNA, leading to the insertion of a gene sequence known as transgene from another plant or a different source, has raised the question of whether transgenic nucleic acids, either DNA or RNA, could be packaged into and/or associated with PDEVs. Indeed, this issue could be relevant for understanding whether PDEVs could represent a vehicle for the diffusion of transgenic DNA into the environment, which could represent a risk if the transgene, as in our case, encodes for antibiotic resistance. Moreover, it could also be a relevant issue if the plant has been engineered to produce therapeutic molecules for biopharming purposes

In this study, RT-PCR was used to investigate the immunomodulating effects of PDEVs on the THP-1 cell line. The results showed that both PDEVs isolated from wt and transgenic tobacco have little anti-inflammatory effects on the cells of the innate immune system. This observation is in line with a previous observation that PDEVs isolated from different sources have anti-inflammatory properties. However, the ability of PDEVs to modulate the immune response appears to be dependent on the type of plant and possibly on the isolation procedure, so further studies are needed to unravel their biological effect. In addition, no significant difference was observed in our model between PDEVs from wt or transgenic tobacco. However, it is not possible to rule out that the immunomodulatory effect of PDEVs may be, in transgenic plants, also dependent on the type of transgene.

In conclusion, our study provides evidence that RNA, but not DNA, could be co-isolated with PDEVs, thus indicating that vesicles protect mRNA, including transgenic mRNA, from degradation, contributing to its cross-kingdom spreading, raising safety concerns, but also opening up the possibility of using PDEVs as a tool to deliver RNA for therapeutic purposes.

## Figures and Tables

**Table 1:** qRT-PCR analysis of NPT-II gene expression in transgenic tobacco. RNA was extracted either by leaf or PDEVs (100 K fraction), with Trifast (method 1) or miRVAna (method 2), and r-verse-transcribed into cDNA, used to amplify the NPT-II gene. The C_t_ of the amplification and the T_m_ of the amplification products are reported. Values are the mean ± SE of three experiments.

	RNA Source for cDNA Synthesis	
	Leaf	PDEVs
RNA extraction method 1		
C_t_	30.08 ± 0.6	33.8 ± 0.3
T_m_ (°C)	89.1 ± 0.1	89.1 ± 0.1
RNA extraction method 2		
C_t_	28.3 ± 0.2	33.7 ± 0.4
T_m_ (°C)	89.1 ± 0.1	89.1 ± 0.1

## Data Availability

The data presented in this study are available on request from the corresponding authors.
